# Farewell Wilfred

**DOI:** 10.1111/1751-7915.12332

**Published:** 2015-10-20

**Authors:** Ken Timmis, Juan Luis Ramos, Willem de Vos, Willy Verstraete, Marty Rosenberg, Siegfried Vlaeminck

It is with great sadness that we announce the untimely death of our friend and colleague, Wilfred Röling, on September 25th. Wilfred was a truly inspirational scholar active in a range of topics within environmental microbiology and microbial biotechnology, and was one of only a handful of researchers worldwide able to effectively combine wet and *in silico* science. The loss to scholarly endeavour of his enormous potential is tragic. His dedication and generosity as an Editorial Board Member of this Journal was exemplary, and his reviews have seriously elevated the quality of many submitted papers, accepted or not by the Journal. He was one-of-a-kind and we shall all miss him and his council. Our thoughts are with his family, friends and colleagues at this difficult time.

